# Barriers to correct pronoun usage in healthcare settings

**DOI:** 10.1186/s12909-024-06032-7

**Published:** 2024-09-27

**Authors:** Jodie Makara, Roman Cain, Lucas Glock, Michael Ioerger, Scott Holliday

**Affiliations:** 1 Department of Physical Medicine and Rehabilitation, University of Washington, Seattle, WA 98195 USA; 2 College of Arts and Sciences, The Ohio State University, Columbus, OH 43210 USA; 3 College of Medicine, The Ohio State University, Columbus, OH 43210 USA; 4 Department of Family & Community Medicine, The Ohio State University, Columbus, OH 43210 USA

**Keywords:** Pronouns, Medical education, Transgender or nonbinary, Barriers to care, Patient–physician relationship, Healthcare experiences, Trans-inclusive healthcare

## Abstract

**Background:**

Using correct pronouns is an impactful way to establish affirming environments for transgender and nonbinary (TGNB) patients. However, physicians often report struggling with this.

**Objective:**

This study set out to conduct an initial root cause analysis of factors contributing to medical students and physicians failing to use TGNB patients’ correct pronouns.

**Methods:**

A 10-item Qualtrics survey was sent to medical students, residents, and physicians practicing in Central Ohio. Participants were asked to describe perceived challenges or barriers colleagues have regarding correctly using TGNB patients’ correct pronouns. A directed content analysis of participant responses was performed utilizing a fishbone diagram root cause analysis tool as a basis for conceptualizing and categorizing barriers. All coding was completed by independent reviewers utilizing a consensus reconciliation methodology.

**Results:**

Of 928 survey respondents, 763 met the study inclusion criteria, of which 453 provided analyzable responses. Of these 453, attendings with five or more years of practice (32.5%) and medical students (27.4%) made up the two largest demographic categories. 1.7% of respondents identified as transgender, nonbinary, and/or genderqueer, and 64% identified as heterosexual/straight. Five core barrier categories were identified: documentation, patient care, environment, knowledge, and individuals. Sub-categories were also identified, including lack of documentation, discomfort, medical culture, lack of standardization, prejudice, and assumptions.

**Conclusion:**

The study identifies important barriers to medical professionals correctly using TGNB patients’ pronouns. The root cause analysis conducted as part of this study demonstrates the necessity of multi-pronged, system-level interventions to support ensuring TGNB patients are addressed using the correct pronouns.

**Supplementary Information:**

The online version contains supplementary material available at 10.1186/s12909-024-06032-7.

## Introduction

More than 1.6 million Americans identify as transgender or nonbinary (TGNB) [1]. While these identities may have unique meanings to specific individuals, transgender and nonbinary people tend to share the commonality of not identifying with the specific binary male or female gender assigned to them at birth. Additionally, nonbinary people (sometimes concurrently identifying as genderqueer) often identify with gender(s) and pronouns outside of the traditional binary. Cultural [2, 3] and linguistic [3, 4] factors contribute to the marginalization and mistreatment of TGNB people within the health care environment [5]. 

Among the LGBTQ + community, TGNB people are at the highest risk for mistreatment and discrimination by health care providers [4, 6]. When seeking medical care, TGNB patients commonly experience feeling misunderstood and disrespected, or are denied care entirely [7–10]. Many factors contribute to this experience for TGNB patients, however, lack of knowledge [9, 11] and exposure [11] are often identified as reasons physicians don’t feel comfortable providing care to TGNB patients. Physicians similarly struggle with the basic skill of using TGNB patients’ correct pronouns. Nonbinary patients are at especially high risk of inaccurate and inconsistent pronoun usage [12]. Improvement in this skill seems like a simple, impactful, no-cost way to create a safe and welcoming environment for TGNB patients [10, 13], but it is often identified as one of the hardest interventions to learn and implement [14, 15]. 

As of 2022, medical schools provided 11 h [16], on average, of LGBT content throughout the entire four-year curriculum, up from 5 h in 2011 [17]. During this time, trans-specific education has increased in some medical schools, but this education is mostly geared towards medical students (not existing practitioners) and is not mandatory [18, 19]. Residents in most programs receive minimal or no trans-specific training, and the unique health concerns of nonbinary/genderqueer patients are rarely discussed [18–20]. Furthermore, many of the educational interventions that are implemented lack specific training on clinical communication with TGNB patients [19]. This lack of education and training is a key barrier and it is commonly identified as the primary reason physicians often make mistakes or neglect to use their TGNB patients’ correct pronouns [13, 14], despite anecdotes and documented patient and clinician experiences suggesting many other factors likely contribute [4, 7, 8]. 

In this study, we used a cross-sectional survey to elicit factors contributing to medical students and physicians not using their TGNB patients’ pronouns correctly and consistently. The goal of this study was to conduct a root cause analysis that will serve as a foundation for developing more effective interventions to ensure TGNB patients are consistently being addressed with the correct pronouns by medical students and physicians. To accomplish this goal, we analyzed and coded responses using directed content analysis methodology. Based on existing literature, the authors anticipated that knowledge, experience, and training would all be reported factors contributing to errors in pronoun usage. We also anticipated that physicians and medical students would identify additional systems factors in other domains that serve as barriers to themselves and their colleagues in addressing TGNB patients with the correct pronouns. Given this, and its effectiveness as a root cause analysis tool in health care settings [21, 22], we utilized the fishbone diagram as a tool to provide a framework for conceptualizing and categorizing barriers during the content analysis process.

## Methods

### Recruitment

This IRB-approved survey was sent to medical students at the Ohio State University College of Medicine (OSUCOM) and Ohio University Heritage College of Osteopathic Medicine (OUHCOM)-Dublin Campus, as well as practicing physicians in Central Ohio (Franklin, Union, Delaware, Licking, Fairfield, Pickaway or Madison counties) using a snowball sampling method. On June 27th, 2022, we sent an initial recruitment email to potential participants using listservs accessible to the research team. We asked participants to forward the survey to other medical students and physicians in the Central Ohio area who might be eligible to complete it. The survey was kept open for one month and a reminder email sent a week before the survey closed on July 27th, 2022.

### Eligibility criteria

People who train or work at a hospital system in central Ohio (including Franklin, Union, Delaware, Licking, Fairfield, Pickaway or Madison counties) as a medical student, resident, fellow, or attending were eligible to complete the survey. PhD faculty in the College of Medicine, other adjacent healthcare professions, or people who practice outside central Ohio were not eligible.

### Survey design

Eligible participants accessed a 10-item, Qualtrics survey including a mix of quantitative and qualitative questions. The lead researcher (JM) and one co-researcher (MI) worked together to develop the survey. JM brainstormed several questions that were then revised down to 8 to promote more participation. There were two questions developed to assess previous training on TGNB patient pronouns, and how prepared individuals felt they were to engage with TGNB patients. 2 questions with two parts each evaluated both individual’s, and perceived colleague’s, self-efficacy for consistently using both their patients’ and coworkers’ correct pronouns. Three questions assessed demographic info of respondents. The free-response question that was the basis for the root cause analysis asked respondents to “please describe any challenges or barriers you have observed to physicians and/or medical trainees correctly using a TGNB patient’s pronouns?” This question was intentionally constructed to encourage respondents to openly share their observations, while minimizing social desirability bias, by asking participants to report any observed barriers without having to identify themselves or others. No pilot testing was completed. However, the questions were reviewed by the research team and a few of JM’s colleagues for clarity and face validity. All responses were kept anonymous and identifying information, such as IP addresses, was not collected. Informed consent to participate was obtained from all participants in the study. See supplemental material for full survey question list.

### Qualitative analysis

To protect the privacy of individuals and organizations in this study, JM exported survey responses from Qualtrics to a password-protected spreadsheet including only response IDs and barriers free-responses. Free-responses were then reviewed by JM to remove individually identifying information. This process resulted in 3 responses being modified: 2 to remove a hospital name and 1 to remove an individual identifier.

**Fig. 1:**
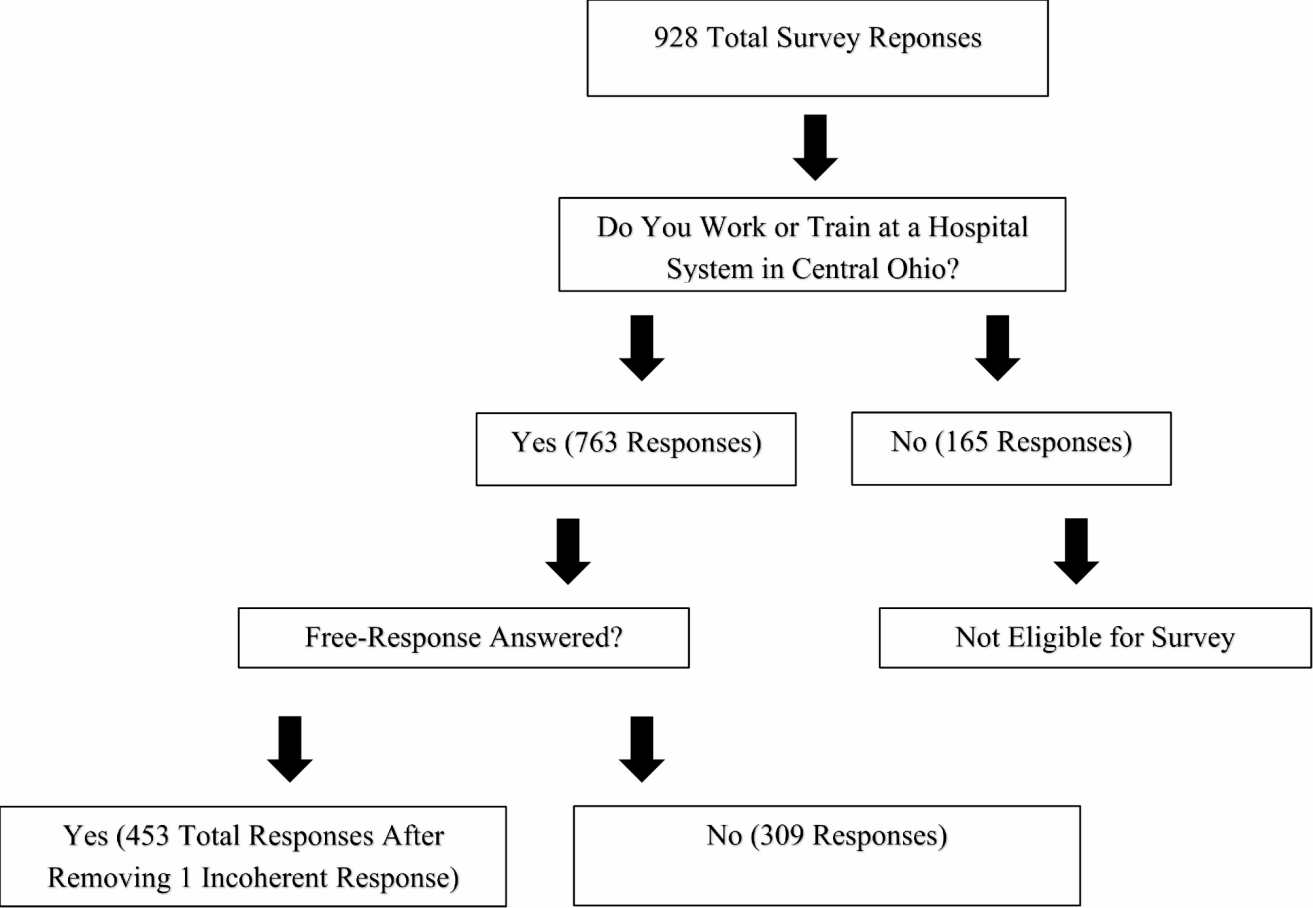
Flow chart of eligible participants

**Fig. 2:**
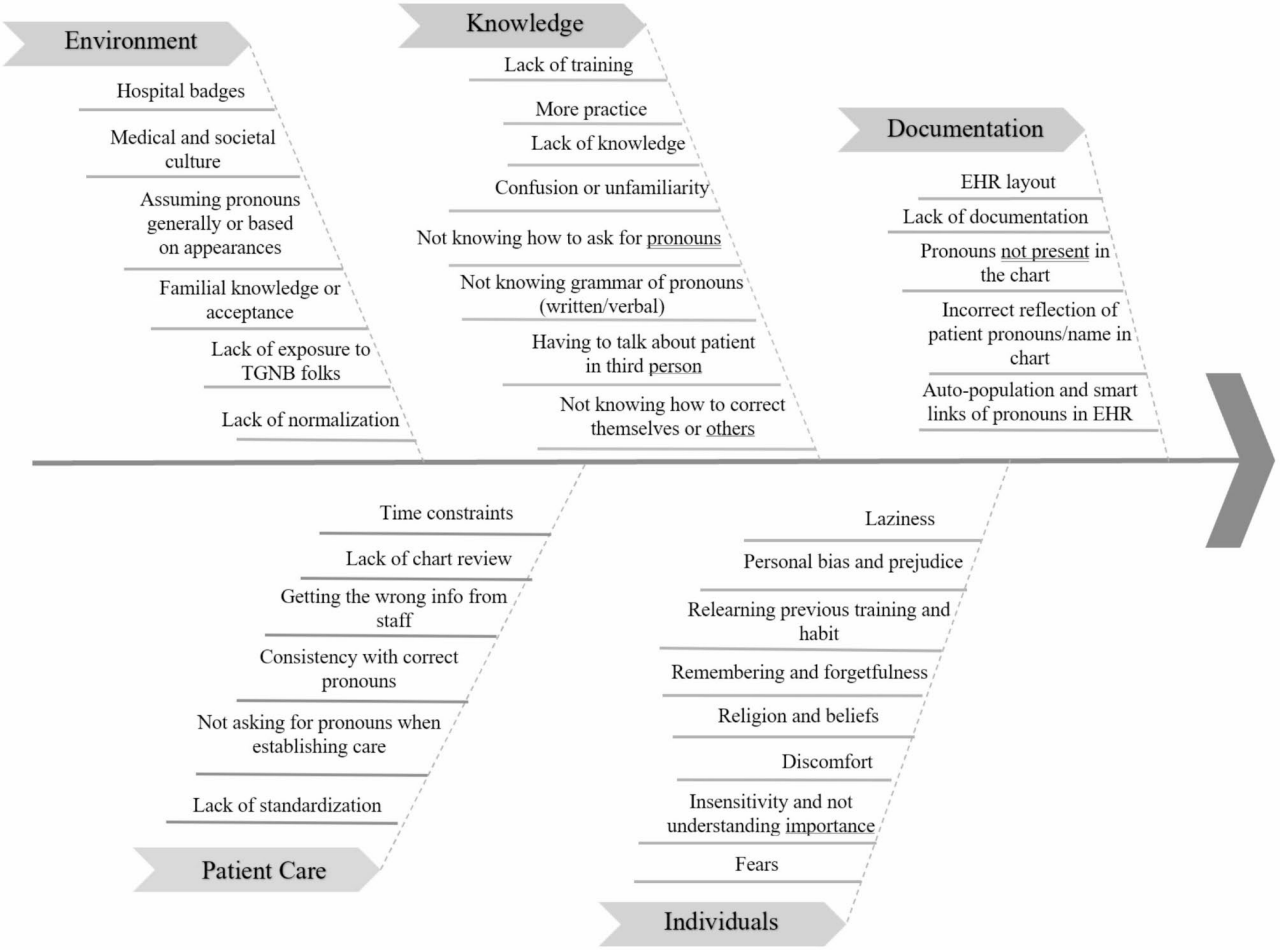
Fishbone diagram of categories and sub-categories of barriers to using correct pronouns

Utilizing a directed qualitative analysis methodology, responses were initially sorted into the 5 categories that typically form the foundation of fishbone diagrams in a root cause analysis: materials, methods, environment, equipment, and people [23]. However, these categories were revised inductively based on the primary root cause themes that emerged from participant responses. The result after two iterations of response review was 5 final primary categories: knowledge, individuals, environment, patient care, and documentation. Additional sub-categories were inductively identified and are highlighted in Fig. 2.

To help ensure accuracy and consistency in coding, all three coders (JM, RC, LG) reviewed 10 sample responses and discussed them together as part of an initial calibration and category definition test process. Every response was then independently reviewed by at least two coders. After each coder independently reviewed and categorized each response, a consensus process was utilized to determine the final categorization for each response. If consensus was not initially reached between the two coders, then the third independent coder helped arbitrate the discussion. Because many responses listed multiple barriers, each individual free-text response could be categorized into one or more categories. At the conclusion of the coding process, all responses and their categorizations were reviewed again to evaluate for any inconsistencies in coding. The three coders (JM, RC, and LG) were the only researchers involved in developing the primary categories listed above.

## Results

There were 928 respondents, 763 of which were eligible based off their profession and region of practice in Central Ohio. Of those, a total of 454 participants (59.5%) filled out the barriers free-response question, with 453 responses used in qualitative analysis and one response being removed consisting of only random letters (see Fig. 1).

The demographic information for eligible survey respondents (*N* = 763) for both participants who provided a free-response answer and for participants who did not provide a free-response answer is presented in Table 1. Due to a high proportion of participants who did not provide a free-response answer also not providing responses to demographic questions, statistical analyses to evaluate for differences between the make-up of the two groups were not conducted. Among the participants who provided a free-response and were thus included in this qualitative analysis presented in this study, the largest subgroups were attending physicians with five or more years of experience (31.8%), medical students (27.4%), and residents (16.3%). The majority of respondents who answered the free-response question reported identifying as heterosexual (64.0%), but 13.2% identified with a non-heterosexual/straight sexual orientation. Additionally, 36.2% reported identifying as male, 50.3% reported identifying as female, and 1.7% respondents reported identifying as transgender, nonbinary, or genderqueer.

**Table 1:** Demographics of eligible survey respondents

*Variable*	*Frequency*			
	*Free-Response Answered*		*Free-Response Not Answered*	
	*N*_*1*_ = 453	*% of N*_*1*_	*N*_*2*_ = 309	*% of N*_*2*_
Level of Training				
Medical Student	124	27.4	69	22.3
Resident	74	16.3	19	6.1
Fellow	25	5.5	13	4.2
Attending (1–4 years of practice)	64	14.1	20	6.5
Attending (5 + years of practice)	144	31.8	47	15.2
Did not provide a response	22	4.9	141	45.6
Previous TGNB Training				
Yes	233	51.4	110	35.6
Yes, but I do not remember	37	8.2	32	10.4
No	183	40.4	139	45.0
Did not provide a response	0	0	28	6.2
Gender Identity				
Male	164	36.2	69	22.3
Female	228	50.3	86	27.8
Trans male	1	0.2	0	0
Trans female	0	0	0	0
Non-binary	6	1.3	0	0
Genderqueer	1	0.2	0	0
Agender	2	0.4	0	0
Questioning	1	0.2	0	0
Something else – please specify	2	0.4	1	0.3
Decline to answer	13	2.9	4	1.3
Did not provide a response	35	7.7	149	48.2
Sexual Orientation				
Asexual	1	0.2	0	0
Bisexual	19	4.2	6	1.9
Pansexual	2	0.4	2	0.7
Lesbian or gay	24	5.3	6	1.9
Heterosexual or straight	290	64.0	126	40.5
Queer	10	2.2	2	0.7
Questioning	4	0.9	0	0
Something else – please specify	2	0.4	0	0
Decline to answer	14	3.1	4	1.3
Did not provide a response	87	19.2	163	52.8

### Categories & sub-categories of barriers to correct Pronoun Use

The categories and sub-categories of barriers to correct pronoun use are discussed below and summarized in the fishbone diagram in Fig. 2. Additional details on the number of respondents, percentage of responses captured by each sub-category, and sub-category exemplary quotes can be found in Table 2.

**Table 2:** Survey quotes representing barriers to correct pronoun usage

Category	Subcategory	Numbers	Percentages (Sub/Cat)	Exemplar Quote(s)	
Knowledge 39.5% (179/453)	More practice	12	6.7% (12/179)	“It can be hard even for people with the best intentions because it takes time and practice to rewire the way our brains have been trained to think about gender.”	
	Lack of knowledge	12	6.7% (12/179)	“…lack of knowledge/training about not assuming pronouns…”	
	Not knowing how to ask for pronouns	44	24.6% (44/179)	“I think the challenges come in not asking for their pronouns, and just finding a formal way to ask about pronouns then integrating that into patient encounters” “…knowing how to broach the topic of people being transgender/nonbinary and how to ask them how they want to be addressed without it coming across as awkward.”	
	Having to talk about patient in third person	14	7.8% (14/179)	“Most of the time in my experience, correct pronoun usage happens not directly in front of the patient, because pronouns are required when referring to someone in the third person.”	
	Not knowing how to correct themselves or others	13	7.3% (13/179)	“I have observed that people feel awkward when [pronouns are] not correct and do not seem to know how to apologize and move on in the future.” “I have had several colleagues that know a patient’s preferred pronouns but still fail to use the correct ones during rounds…I have kindly reminded them but it doesn’t always work.”	
	Lack of training	23	12.9% (23/179)	“We had brief training, but it wasn’t comprehensive.”	
	Not knowing grammar of pronouns (written/verbal)	28	15.6% (28/179)	“Have difficulty saying unique pronouns. Some find it very challenging incorporate a new word when you do not know how to pronounce it or do not understand the spelling. Much easier to interchange she vs he vs they than more unique pronouns.” “It’s altering an entire facet of our language by adding new words (eg, ze/zirs) and changing the rules of grammar entirely (eg, using they/them to refer to a single person rather than as a pleural pronoun). It’s like learning a new language…”	
	Confusion or unfamiliarity	50	27.9% (50/179)	“Lack of understanding of the appropriate ways to ask individuals about pronouns…” “Being unfamiliar with how to use pronouns other than he/him/his or she/her/hers, forgetting or unconsciously using incorrect pronouns.”	
People 31.8% (144/453)	Discomfort	40	27.8% (40/144)	“Some clinicians seem to feel uncomfortable asking people what their preferred pronouns are.” “[Toxic language] makes me uncomfortable to bring up my own pronouns” “Find it sometimes uncomfortable to incorporate into conversation”	
	Remembering and forgetfulness	29	20.1% (29/144)	“I’ve seen physicians and trainees forget a transgender or nonbinary patient’s pronouns and accidentally misgender them.”	
	Fears	26	18.1% (26/144)	“Worries about making mistakes and inadvertently using the wrong pronouns… Worries about criticisms that can arise from the above mistakes or jeopardizing patient relationships.” “I think just not wanting to offend anyone but also respecting their pronouns…” - “…it is ideal to consistently ask all patients for their pronouns but in some populations asking for pronouns confuses patients (older, neurocognitively impaired, perception that asking would upset a patient due to political opinions).”	
	Personal bias and prejudice	18	12.5% (18/144)	“I’ve observed and had to directly address staff members (nurses/techs/etc) who have intentionally not used the patients pronouns. I’ve seen people roll their eyes, say things like “oh look at you being all politically correct” and other condescending things when using a patients correct pronouns…but there is definite bias against our patients and working with a staff that is not accepting can certainly make it harder to do the right thing,” “…The other big challenge is some folks just don’t care and actively are transphobic and will engage in dead-naming and intentionally using the sex-assigned at birth”	
	Insensitivity and not understanding importance	29	20.1% (29/144)	“…there is an extra measure of respect required to ensure you are using the correct pronouns when you are speaking with family or other practitioners, since the stakes might not seem as high since you are not in front of the patient.”	
	Laziness	6	4.2% (6/144)	“I have seen physicians who don’t want to take the time to understand what this “new” phenomenon is about, even though trans and non-binary people have always existed.” “lack of motivation”	
	Relearning previous training and habit	23	16.0% (23/144)	“There is still a subconscious pattern we all have to make snap use of pronouns without thought in conversations…It takes effort to avoid [labeling by looks] that we just innately do when discussing patients who are not transgender” “I think even well-intentioned folks who truly believe in the importance if using personal pronouns struggle to overturn years of practiced behaviors and will default to he/him, she/hers and just use the assigned sex at birth…” “I was trained to let the patient introduce themselves with the name and pronouns they prefer. Many other physicians seem to be trained to assume the patient goes by Mr/Ms Lastname…” “I find non-binary pronouns more difficult to use personally just from a habit of using binary pronouns in everyday speech my whole life”	
	Religion and beliefs	10	6.9% (10/144)	“God does not make mistakes….He made men and women…since you cant’ be both, the medical center is wasting a lot of time and resources on a non-issue. I would like the medical center to focus on safe patient care and stop the “wok” nonsense.” “As a person of faith myself, whose belief system celebrates the beauty of men and women and recognizes them as such based on chromosomally assigned sex while celebrating the entire range of masculinity and femininity natural to an individual regardless of sex, I deal with this myself…”	
Environment 26.9% (122/453)	Lack of exposure (to TGNB folks)	24	19.7% (24/122)	“For many this is an area that they have little experience or education.” “The greatest challenge is the years of binary norms that have been established - growing up in the 80’s and early 90’s, I had no exposure to non-binary individuals or the utilization of non-binary pronouns.”	
	Assuming pronouns generally or based on appearances	49	40.2% (49/122)	“…if the patient’s presentation is more ambiguous, or the presentation may be perceived as more congruent with a gender other [than] the preferred gendered pronoun.” “Often I think people (myself included) use the pronoun we presume the person we are referring to or speaking with would use based on the person’s external appearance…”	
	Lack of normalization	17	13.9% (17/122)	“Trainees not introducing themselves with pronouns but that is because the current environment/ practice has not been to introduce with gender pronouns (even within faculty).” “I think the biggest challenge or barrier I have observed is simply that it is not yet standard to offer one’s own pronouns in the medical field, or to ask for every patient’s pronouns.”	
	Familial knowledge or acceptance	17	13.9% (17/122)	“As a pediatrician it is important to understand if the patient’s parent/caregiver is aware of/ supportive of their pronouns, and I don’t want to risk disclosing something inadvertently.”	
	Medical and societal culture	37	30.3% (37/122)	“Creating environments of inclusion is definitely a barrier that exists. Many patients are scared of sharing their pronouns or preferred name due to rampant stigma that exists in the healthcare field (and beyond), and are therefore not receiving adequate care.”	
	Hospital badges	2	1.6% (2/122)	“I think that [adding pronouns] to the badges would make attendings more aware”	
Patient Care 21.6% (98/453)	Not asking for pronouns when establishing care	20	20.4% (20/98)	“Not initially knowing the patient’s preferences (and not knowing they have a specific preference) when I begin my interaction with them.”	
	Lack of chart review	11	11.2% (11/98)	“Misgendering upon first introduction due to lack of chart attention by physician or trainee.”	
	Consistency with correct pronouns	24	24.5% (24/98)	“I have seen physicians / medical professionals and trainees having difficulty approaching how to ask someone for their pronouns and sometimes even mixing them up as they are talking to them. Most apologize quickly and reset, however that does not always happen.” “I’ve seen residents get it right with the patient and wrong in the workroom.” “I think in medical presentations [the pronoun] sometimes gets mixed up”	
	Time constraints	24	24.5% (24/98)	“Physicians and trainees may have limitations in time that do not allow them to ask a patient’s chosen pronouns.” ““it is not always feasible to ask [for pronouns] in critical or time-restricted environments.”	
	Getting wrong info from staff	14	14.3% (14/98)	“The ancillary staff generally have the best opportunity to identify and select an individual’s pronouns in the electronic medical records during intake. If these are not selected correctly or the ancillary staff does not have adequate training to ascertain this information, then the bias becomes codified in the system.”	
	Lack of standardization	19	19.4% (19/98)	“It is not common practice to ask ALL patients what their preferred pronouns are when initiating a patient-provider relationship… This should be more in our script for all our encounters and not something that is just assessed once and added in the medical chart, but that is assessed with all our new relationships (both personal and professional).” “IF the faculty do not introduce themselves with pronouns, I can see why students/trainees would not as well.”	
Documentation 20.5% (93/453)	Lack of documentation	22	23.7% (22/93)	“The chart generally tells us assigned sex, not preferred gender pronouns” “Not noted in Med records, only a few options in the system versus the numerous pronouns those such as gender fluid, genderqueer, or gender nonbinary identify as”	
	Incorrect reflection of patient pronouns/name in chart	32	34.4% (32/93)	“There are often incongruities in charting about a patients preferred name/gender/pronouns”	
	Pronouns not present in the chart	14	15.1% (14/93)	“Sometimes the pronouns in the chart are not listed, or the chart lists sex assigned at birth as well as gender with no pronouns”	
	Auto-population and smart links of pronouns in EHR	11	11.8% (11/93)	“EMR refreshable smart links that use only gender associated with sex assigned at birth rather than pulling the pronoun preference makes it difficult because it reinforces non preferred pronouns in writing”	
	EHR layout and ease of finding info	31	33.3% (31/93)	“EMR does not make it obvious. Sometimes, patient is entered in as their birth gender. Would be super helpful if the EMR included pronouns and this was standard at intake” “Sometimes it is not readily visible or easy to find in the chart if the patients preferred pronouns do not agree with their legal name in the chart.”	
No Barriers		40	----	“none” “n/a” “no barriers” “have not observed any barriers”	

#### Knowledge

Barriers related to knowledge were mentioned by 39.5% of respondents. Sub-categories that were identified included: needing more practice, not knowing how to ask for pronouns, not knowing how to correct themselves or others, not knowing grammar of pronouns (written/verbal), and confusion or unfamiliarity. The overarching message across responses in these sub-categories was that respondents believed the amount or scope of training they have received had not adequately prepared them to know how or when to ask for pronouns nor understand how to use pronouns correctly in sentences. Respondents felt they have not been given the tools necessary to practice and improve comfort and competence with using pronouns. Respondents also felt without repetition and training, they feel less prepared when interacting with TGNB patients.

#### Individuals

Barriers related to people were mentioned by 31.8% of respondents. Responses in this category mainly discussed cognitive or psychosocial barriers of the individual, like feelings, beliefs, and personality. Sub-categories that were identified included: discomfort, remembering or forgetfulness, fears, personal bias/prejudice, insensitivity or not understanding importance, laziness, relearning previous training/habit, and religion or beliefs. The overarching message across responses in these sub-categories was that respondents believed there are numerous intra- and inter-personal struggles that make it challenging to ask for and use patient’ correct pronouns, such as general discomfort, fear, prejudice, and religion. These topics require conversations that go beyond basic terminology, grammar, and general awareness. Respondents felt these are complex issues, including components of misinformation, that need to be addressed in trainings to better prepare them to navigate these scenarios in health care settings and be better allies to TGNB patients.

#### Environment

Barriers related to environment were mentioned by 26.9% of respondents. Responses that talked about other people or situations impacting one’s ability to use a patient’s correct pronouns were placed in this category. While there may be some overlap between categories, responses were included here if it felt like they were addressing the impact of others as opposed to inherent qualities of individuals. Sub-categories that were identified included: lack of exposure (to TGNB folks), assuming pronouns generally or based on appearances, lack normalization of asking and sharing pronouns, familial knowledge or acceptance, medical and societal culture, and hospital badges. The overarching message across responses in these sub-categories was that respondents believe having an unsupportive environment makes it challenging to do the right thing, even if it’s what they were taught. Respondents express that if the people they work with shared the same intentions to ask all patients for their pronouns and were not met with resistance from other staff members, then they would feel more comfortable doing it themselves. Another significant challenge noted by respondents is when they are unsure whether the patient is out or accepted by those around them, it feels difficult for them to ask or use the correct pronouns in these scenarios.

#### Patient care

Barriers related to patient care were mentioned by 21.6% of respondents. Responses in this category discussed process errors in patient care, that if corrected, would lead to more seamless use of correct patient pronouns. Sub-categories identified included: not asking for pronouns when establishing care, lack of chart review, consistency with correct pronouns, time constraints, getting wrong info from staff, and lack of standardization. The overarching message across responses in these sub-categories was that respondents believe having little time and no set script when interacting with patients makes it challenging to acquire the correct info on pronouns, especially when other staff members contribute to misinformation. They suggest that if they had a more standardized method to collect this information and model physicians putting it into practice more regularly, then physicians would be less likely to mix up pronouns for patients.

#### Documentation

Barriers related to documentation were mentioned by 20.5% of respondents. Responses in this category mainly discussed the electronic health record (EHR) and ease of navigating it. Sub-categories that were identified included: lack of documentation, pronouns not being present in the chart, incorrect reflection of patient pronouns/name in the chart, auto-population and smart links of pronouns in EHR, and ease of finding info within EHR layout. The overarching message across responses in these sub-categories was that respondents believe the lack of visibility of patients’ correct name and pronouns on paper and in the EHR makes it challenging to use patients’ correct name and pronouns. They suggest that, if the tools being used show consistent pronouns, then physicians will have an easier time staying consistent in addressing patients correctly.

#### No barriers

Responses that did not mention barriers included 9% of respondents and explicitly stated things like “none,” “n/a”, or “have not observed any barriers.”

## Discussion

Using TGNB patients’ correct pronouns is a quick, free and impactful way to create a safer environment for TGNB patients [4, 13]. However, it is not easy to implement consistently [14, 15]. In this study, we set out to understand the barriers medical students and physicians perceive with respect to using TGNB patients’ correct pronouns. As anticipated, knowledge, training, and experience were barriers mentioned in many of the responses (39.5%). However, documentation, individual provider factors, the patient care process, and the patient care environment also emerged as major domains in which many barriers exist. Thus, this study highlights the impact of system-level factors on correct pronoun usage, with respondents reporting that it can be challenging even for knowledgeable, motivated, and skilled individuals to consistently use correct pronouns with their patients if they are in unsupportive environments and systems. For example, participants reported their electronic medical records are designed in a way that does not make it easy to input or look up patient’s correct pronouns and include them in notes. In this instance, physicians may know they need to use correct pronouns and have the comfort and experience to do this seamlessly and effectively, but they may still fail to use patients’ correct pronouns in conversation and in their notes because of structural issues. As such, this study highlights many other psychosocial, intrapersonal, and structural factors beyond knowledge, skills, and experience that contribute to physicians not consistently addressing TGNB patients with the correct pronouns.

### Using root cause analysis to inform interventions

In this study, a fishbone diagram was utilized because it provides a simple, visual framework for organizing a root cause analysis to identify potential areas of breakdown that can contribute to an overall outcome. In addition to highlighting numerous novel and familiar barriers to using correct pronouns, this study highlights how intertwined the barriers are that impact physicians’ behavior. Interventions to support correct pronoun use often focus on trying to create awareness, build knowledge, and change attitudes [19]. However, this study demonstrates that there are many factors that can prevent the use of correct pronouns, even if physicians are aware of the importance and are committed to doing so. The following are a few examples of how the barriers identified in this root cause analysis can be used to inform interventions, with additional examples of ideas provided in Table 3.

**Table 3:** Educational interventions for barriers

Barriers	Education Interventions
Lack of documentation Incorrect reflection of patient pronouns/name in chart Pronouns not present in the chart EHR layout and ease of finding info	• All physicians and medical staff should be trained on where and how to input patient name, pronouns and gender identity into the EHR. • Make pronouns readily visible/accessible for providers to see and edit as necessary (i.e. EHR sticky notes and large banner flags) • Teach providers how to utilize EHR smart phrases to help input patient pronouns easily into notes. • Utilize three gender fields in EHR demographics including gender ID, birth sex, and legal sex to help identify transgender patients and utilize asterisks or alert icons when patient gender ID and legal sex do not align or patient uses a different name than their legal name [29] • Use existing online training tools to expand institutional knowledge of gender ID data collection in clinical settings, such as the Center of Excellence for Transgender Health at the University of California, San Francisco (http://transhealth.ucsf.edu/video/story.html) [29] • Avoid using “other” as an alternative gender option, instead provide a wider variety of gender identities for patients to choose from or include a “fill-in-the-blank” option [30] • Use validated scales and psychometric measures of discrimination to identify structural improvement in the EHR to improve safety, comfort, and access to TGNB affirming healthcare [42].
Lack of standardization Not knowing how to ask for pronouns Not knowing how to correct themselves or others Discomfort	• Provide standardized language such as, “How would you like me to address you today?” or “Is it okay if I use these pronouns for you?” • Institutions should make it in expectation to acquire pronouns at the start of every patient encounter • Medical schools should include collection of pronouns and gender identity of standardized patients in all simulated cases, not just those that are explicitly known to have transgender patients or patients with HIV or other STIs to avoid reinforcing harmful stereotypes • Training needs to include how to support a TGNB person when misgendering happens (i.e. acknowledging the unpleasant interaction, asking how they can advocate for them in the future) [43] • Name the discomfort and provide space for vulnerable discussion and self-examination of the causes, [41, 37]
Lack of normalization	• Understanding the importance of holding physicians and others accountable • Place greater emphasis on institutional guidelines and standards of care [24] that explicitly address respecting patient pronouns and identities [4]
Familial knowledge or acceptance	• Understand that patients may be at different levels of coming out and approaches for asking about pronouns will be unique to the situation • Include clinical vignettes that discuss intrafamilial relations and how to support a patient when they are surrounded by people who do not respect their identity
Lack of exposure (to TGNB folks)	• Provide qualitative literature of first-hand experiences from TGNB patients and physicians [10, 44, 45] • Collaborate with transgender and nonbinary healthcare professionals who are willing to teach and provide adequate compensation [18] • Incentivize and support role models [46] who support the development of competent professionals • Improve efforts to hire TGNB medical staff for greater visibility so TGNB trainees and patients have mentors and providers who look like them, expanding safe spaces and allowing cisgender trainees to learn from those willing to teach on their lived experience
Insensitivity and not understanding importance	• Include rationale behind adding more inclusive questions to intake forms/having pronouns on hospital badges • Discuss the difference between generalizations and stereotypes (while the former opens doors to thoughtful care, the latter closes them) [41]
Lack of training Remembering and forgetfulness	• Provide easily searchable digital access to previous trainings on correct pronoun usage. Create a database of information for interested physicians and others to access all information from previous trainings to continue solo practice, with a place for remote questions to be addressed • Provide access to a clinician champion who can be contacted with questions related to using correct pronouns for patients. • Provide and publicize annual opportunities for brief trainings on using correct pronouns with patients. Education should be longitudinal [33] with the understanding of “use it or lose it” when it comes to asking patients for their pronouns • Utilize roleplay with trans standardized patients when possible [18]
Lack of knowledge Confusion or unfamiliarity	• Collaborating with local LGBTQ organizations for educational materials and speakers to get non-physician perspectives to avoid putting unnecessary stress on patients to educate their providers. • Utilize LGBT provider resources made available online by national organizations like WPATH [47] or GLMA [48]
Not knowing grammar of pronouns (written/verbal)	• Written and verbal examples of how to use gender-neutral pronouns, including neopronouns, [12] or how to inquire about them respectfully • More discussion of nonbinary identities [5, 6] and language [3] is needed in trainings • Pronunciation of neopronouns (i.e. ze/zir or fae/faer) should be covered in trainings and practiced in conversation and on paper using sentence templates to familiarize staff with less common pronouns
More practice Having to talk about patient in third person Fears Consistency with correct pronouns	• When possible, institutions should incorporate teaching trainees and staff the fundamentals of gender identity, culturally competent pronoun acquisition and continued use correctly into mandatory trainings. • During training sessions, facilitate interactive involvement from participants to gain competency [7, 18] • Support a culture of minimizing judgment for making mistakes, while not letting mistakes go unacknowledged. Minimize expectations of perfection on the first try, progress is what matters [10] • Institutions should minimize use of the data-to-text functionality within EHR systems that creates “gendered” natural language, when inputting physical exam findings for example, which could cause problems for transgender or nonbinary patients whose pronouns may not be a coded option and leads to misgendering [29]
Medical and societal culture Personal bias and prejudice Religion and beliefs	• Incorporate explanations of intersectionality where pronouns and gender identity are part of the patient along with disability status, race, socioeconomic status, and personal background and how all these elements are included in holistic healthcare [43]. • Consider discussion of professional ethics and the need to respect patient’s identities as a commitment to medical practice. • Discuss the ethical dimension of nonmaleficence and misgendering (respecting patient autonomy, dignity, and addressing interpersonal stigma [8] as a cause of psychological harm and inadvertent physician malpractice)

First, a major domain of barriers identified by the respondents in this study are problems related to inaccurate or absent documentation. The World Professional Association for Transgender Health (WPATH) Standard of Care Version 8 [24], WPATH’s Electronic Medical Record Working Group [25], and the Uniform Data System for Health Centers [26] either recommended or mandated the collection of gender identity data in the EHR. Additionally, growing evidence suggests current names and pronouns are equally, if not more important to include [25, 27, 28]. Despite this, our study highlights an overwhelming lack of pronoun documentation in the EHR, often due to unorganized layouts, smart phrase inconsistencies, and simply not acquiring this info to upload into patient charts. Strategies have been proposed to help institutions develop more inclusive data collection, including standardizing the collection of gender ID (otherwise labeled as gender identity), birth sex, and legal sex, so that all three demographic components are used to more accurately identify transgender patients [29]. Additionally, the use of “other” as a third demographic category, which is still commonly used in an attempt to include nonbinary patients, often leads to feelings of literal “othering” and should be replaced with the option to provide more specific identities beyond male and female [30]. These subtle discrepancies in patient report and legal documents needs to be automatically linked and brought to staff and physicians’ attention. Colorful banner display methods in patient charts with pop-up reminders can clarify how the patient would like to be addressed even before the initial encounter [29, 30]. However, protecting patient privacy [24, 30] is also crucial to minimize transphobic terminology, a form of EHR-mediated violence [31]. For example, if patients select “prefer not to disclose,” which should always be offered as an option on intake forms, this could activate a series of privacy options. With that, patients can determine the pronouns they would like used for certain types of healthcare encounters based on their feelings of safety [30]. Unfortunately, our study indicates many of these strategies are not yet being implemented across several different hospital networks. As a result, we suggest educational initiatives need to be paired with advocacy that pushes to change informational systems [32] in ways that challenge assumptions [33], minimize TGNB erasure [32], and facilitate using patients’ correct pronouns.

Second, medicine is a fast-paced environment that pushes people to make assumptions more than they otherwise might like, which contributes to errors in communication and allows unfiltered bias to disrupt the patient-physician relationship. Cisgendered medical organizations perpetuate harmful stereotypes and a hostile environment for TGNB individuals and allies [34]. Medical training requires countless hours of studying, clinical time, and a constant influx of new material to memorize while the hours in a day remain the same. Respondents in our study brought to light various barriers that are a result of having inadequate time in the busy day. These include having little time for formal introductions with new patients to discuss pronouns, and not having time to pre-chart on patients ahead of time. Often it is left up to individual physicians, with their variable levels of fatigue, investment, and competing interests, to take it upon themselves to question their own assumptions and ensure accuracy with their language during patient care [33]. As a result, our participants reported many instances of hesitancy to do the right thing, despite some having acquired knowledge and training due to both individual and systems-level factors. These factors include discomfort, fear, and a lack of normalization around topics of pronoun usage for TGNB patients, in addition to a lack of standardized processes for the collection and utilization of pronouns. Thus, in these fast-paced, cognitively taxing situations, it is important to construct processes, systems, and culture that facilitates using patients’ correct pronouns.

Third, forgetfulness and needing more practice were two other barriers mentioned by many respondents in this study. Other studies have shown physicians [35] and medical students [36] often report increased comfort immediately following training but lose efficacy in their competence the further they get from their training [35]. Our study corroborates these studies given that nearly 1 in 10 respondents disclosed forgetting much of what they had previously been taught in trainings as a primary barrier to correct pronoun use. As such, repetition in curriculum, rather than single, one-off educational experiences are needed to help medical students and physicians retain the knowledge and skills they have learned [20]. This repetition does not need to be time intensive, but rather can be in the form of brief refreshers, to support physicians in making lasting changes. This could include interactive modalities involving TGNB people in case-based discussions or as standardized patients, as well as brief role-playing to provide more opportunities to practice and gain confidence [19]. This practice can be reinforced by making system-level process changes to ensure intake forms and smart phrases prompt a discussion of pronouns. This can help create opportunities in everyday practice to utilize individual knowledge and skills to ensure correct pronouns are being used to address patients.

### Limitations and strengths

Limitations of this study include the subjective nature of qualitative findings and social desirability bias present in survey responses. To minimize the impact of these limitations on the outcomes of this study, the survey promoted anonymity and allowed respondents to complete the survey in a private setting. Additionally, respondents were asked to report what barriers they have observed physicians and medical students face, as opposed to being asked to report on their own potentially negative behaviors. Finally, responses to demographic questions were not required. Many respondents chose not to provide complete demographic information, which limited our ability to conduct certain analyses (e.g., evaluate demographic differences between those who did and did not answer the free-response questions). However, we wanted participants to feel like they could respond freely without worrying about being identified. An additional limitation to our study is that we restricted survey eligibility to medical students and physicians who work in a hospital system given our focus on informing the kinds of interventions used to make changes in these kinds of environments. As a result, the perspectives of PhD faculty, hospital system staff, and other health care providers are missing from this root cause analysis. We also are missing out on the responses from physicians who do not work as part of a hospital system. However, we do believe our results are minimally generalizable to academic medical centers and other large hospital systems, and it is quite likely these results are generalizable beyond these specific environments.

A strength of this study is the large dataset obtained for a qualitative study, providing us with more insight to help target interventions. While choosing to limit responses to those practicing or training in central Ohio could be seen as a limitation, we felt it was a strength because anchoring the recruitment for this study in a specific geographic area allowed the research team to leverage a broader, more diverse recruitment network than what would have likely been possible if this study did not have this local emphasis. By expanding our survey nationally, we might have primarily received responses from individuals in the LGBTQ + community. Using this method of recruitment, we gathered responses from a large proportion of heterosexual and cisgender participants, providing us with categories that should be more generalizable than if we had primarily LGBTQ + respondents. Another strength is utilizing three separate transgender researchers to independently code the data for categories and a consensus reconciliation process to code and clarify categories and subcategories. This methodology allowed for more robust analysis and discussion than otherwise might be seen in studies made up of only cisgender researchers.

### Future directions

This study identifies many barriers that impact the use of correct pronouns by medical students and physicians. Most current trans-inclusive trainings focus on terminology and general etiquette when interacting with TGNB people [17, 18]. On the surface, this seems like the most impactful starting point for creating change to ensure TGNB patients are addressed with the correct pronouns. However, many respondents in our study identified key structural, process, and medical culture issues that serve as barriers to themselves and their colleagues consistently using correct pronouns. As a result, our work suggests creating training, processes, and systems [32] that fit within physician time constraints are needed to support consistent, correct pronoun usage for patients. Additionally, trainings and institutional initiatives will likely need to address the psychosocial issues, such as discomfort [36, 37], fear [9], beliefs [38, 39], cultural cognitive load [40], and assumptions [4, 40, 41] through structural and cultural interventions. Given the current political climate and variability between municipalities and their legislative agendas, it may be challenging in some places to get buy-in for substantive system-level changes. As such, both research and advocacy are needed to determine the most practical and effective combination of training and system-level changes necessary to ultimately influence medical students’ and physicians’ use of TGNB patients’ correct pronouns.

## Conclusion

This study broadly evaluates, categorizes, and unravels the complex interconnectedness of medical students’ and physicians’ perceived barriers to correct pronoun usage. Our study identified barriers related to patient care, documentation of pronouns in the EHR, and the ways our environment makes it challenging to ask for and use TGNB patients’ correct pronouns, in addition to the knowledge and skills typically identified as the primary barriers. As a result, this highlights a number of important process and system-level barriers that need to be addressed to effectively facilitate medical students and physicians using TGNB patients’ correct pronouns accurately and consistently.

## Electronic supplementary material

Below is the link to the electronic supplementary material.

Supplementary Material 1

## Data Availability

The datasets used and/or analyzed during the current study are available from the corresponding author on reasonable request.

## Declarations

### Disclaimers

The views expressed here are those of the authors and do not reflect views of Ohio State University College of Medicine as a whole.

### Previous presentations

Parts of this study were presented at Transforming Care Conference in Columbus, Ohio, October 18th, 2022.

## Abbreviation

TGNB: Transgender or nonbinary
LGBTQ: Lesbian, gay, bisexual, trans, queer
EHR: Electronic Health Record
