# Supplementary material for: Barriers to correct pronoun usage in healthcare settings
Source: BMC Med Educ. 2024 Sep 27;24:1056. doi: 10.1186/s12909-024-06032-7 (PMC11428900; doi:10.1186/s12909-024-06032-7)
Supplement: Supplementary file 1 — Supplementary Material 1 [file 12909_2024_6032_MOESM1_ESM.docx]

*Experience and Observations Questions*

1. “Have you had any training specifically on how to obtain and use a transgender or nonbinary (TGNB) patient’s pronouns?” (Yes; Yes, but I do not remember what I was taught; No)
2. How well prepared do you believe you are to interact with TGNB patients?
    (0 = Not Well Prepared at All, 10 = Very Well Prepared)
3. FREE-RESPONSE: “Please describe any challenges or barriers you have observed to physicians and/or medical trainees correctly using a TGNB patient’s pronouns?”

*Self-Efficacy Questions [These items were ancillary and not the focus of analysis in this study.]*

1. Please indicate the extent to which YOU are confident consistently doing the following actions. (0 = Not Confident at All, 10 = Very Confident)
   1. Using your patients’ pronouns correctly.
   2. Using your coworkers’ pronouns correctly.
2. Please indicate the extent to which you believe YOUR COLLEAGUES are confident consistently doing the following actions. (0 = Not Confident at All, 10 = Very Confident)
   1. Using their patients’ pronouns correctly.
   2. Using their coworkers’ pronouns correctly.

*Demographic Questions*

1. What is your level of training? (medical student; resident; fellow; attending (1-4 years of practice); attending (5+ years of practice)
2. What is your gender identity? (Female; Male; Genderqueer; Agender; Non-binary; Transmale; Transfemale; Questioning; Something Else – please specify (free text); Decline to answer)
3. What is your sexual orientation? (Asexual; Bisexual; Pansexual; Lesbian or gay; Heterosexual or straight; Queer; Questioning; Something Else – please specify (free text); Decline to answer)
